# Beetle bombing always deters praying mantises

**DOI:** 10.7717/peerj.11657

**Published:** 2021-09-03

**Authors:** Shinji Sugiura

**Affiliations:** Graduate School of Agricultural Science, Kobe University, Kobe City, Hyogo Prefecture, Japan

**Keywords:** Bombardier beetles, Brachinini, Carabidae, Chemical defences, Mantodea, Predator, Prey

## Abstract

Some animals have evolved chemical weapons to deter predators. Bombardier beetles (Coleoptera: Carabidae: Brachininae: Brachinini) can eject toxic chemicals at temperatures of 100 °C from the tips of their abdomens, ‘bombing’ the attackers. Although some bombardier beetles can reportedly deter predators, few studies have tested whether bombing is essential for successful defence. Praying mantises (Mantodea) are ambush predators that attack various arthropods. However, it is unclear whether bombardier beetles deter mantises. To test the defensive function of bombing against praying mantises, I observed three mantis species, *Tenodera sinensis*, *Tenodera angustipennis*, and *Hierodula patellifera* (Mantidae), attacking the bombardier beetle *Pheropsophus jessoensis* (Carabidae: Brachininae: Brachinini) under laboratory conditions. All mantises easily caught the beetles using their raptorial forelegs, but released them immediately after being bombed. All of the counterattacked mantises were observed to groom the body parts sprayed with hot chemicals after releasing the beetles. When treated *P. jessoensis* that were unable to eject hot chemicals were provided, all mantises successfully caught and devoured the treated beetles. Therefore, bombing is essential for the successful defence of *P. jessoensis* against praying mantises. Consequently, *P. jessoensis* can always deter mantises.

## Introduction

Prey animals escape from predators in various ways (Edmunds, 1974; Sugiura, 2020a). Prey must evade predators at a stage in the predator behavioural sequence ‘encounter’, ‘detection’, ‘identification’, ‘approach’, ‘subjugation’, and ‘consumption’ (Endler, 1991). Many studies have shown that prey can evade predators before subjugation (Edmunds, 1974; Ruxton, Sherratt & Speed, 2004), and recent studies have indicated that prey can escape predators after subjugation (Umbers & Mappes, 2015; Sugiura & Sato, 2018; Sugiura, 2020a, 2020b).

Many animal species have evolved chemical weapons to defend themselves against predators (Eisner, 1970; Eisner, Eisner & Siegler, 2005). For example, some insects produce or sequester toxic chemicals that prevent predators from swallowing them (Eisner, 1970; Nishida, 2002; Eisner, Eisner & Siegler, 2005). In many chemically defended prey, contact with a predator triggers the ejection of defensive chemicals (Eisner, 2003; Jones & Bulbert, 2020). Because toxic chemicals can damage the predator digestive systems (Whitman & Vincent, 2008; Perelman & Chikatunov, 2010; Sugiura & Sato, 2018), many predators reject the chemically defended prey before swallowing them (Dean, 1980a; Taniguchi et al., 2005; Whitman & Vincent, 2008; Matsubara & Sugiura, 2017; Sugiura, 2018). However, predators with toxin tolerance can eat the chemically defended prey (Dean, 1980a; Fink & Brower, 1981; Sugiura & Sato, 2018). Therefore, the effectiveness of chemical defences depends on the predator species and individuals (Sugiura & Sato, 2018).

Adults of the beetle tribe Brachinini Bonelli (Coleoptera: Carabidae) can eject toxic chemicals at temperatures of approximately 100 °C (i.e., bombing) from the tips of their abdomens in response to a predator attack (Aneshansley et al., 1969; Dean, 1979; Eisner, 2003; Eisner, Eisner & Siegler, 2005; Arndt et al., 2015). Beetles of the subfamily Brachininae Bonelli, which comprises the tribes Brachinini (645 species in 8 genera) and Crepidogastrini Basilewsky (123 species in 6 genera), are called ‘bombardier beetles’ (Arndt, Beutel & Will, 2016; Anichtchenko et al., 2021). Bombardier beetles of the tribe Brachinini store hydroquinone and hydrogen peroxide separately in two reservoirs in the abdomen (Eisner, 2003). When the aqueous solutions of hydroquinones and hydrogen peroxide reach the reaction chamber from each reservoir, enzymes (catalysts) facilitate oxidation of the hydroquinones and decomposition of the hydrogen peroxide (Eisner, 2003). An explosive reaction ejects the reactants and boiling water. Some bombardier beetles can aim the hot chemicals in virtually any direction (Eisner & Aneshansley, 1999). Although many studies have investigated whether bombardier beetles can defend against predators (Eisner, 1958, 2003; Eisner & Meinwald, 1966; Eisner & Dean, 1976; Dean, 1980a; Conner & Eisner, 1983; Nowicki & Eisner, 1983; Eisner, Eisner & Aneshansley, 2005; Eisner et al., 2006; Sugiura & Sato, 2018; Sugiura, 2018; Kojima & Yamamoto, 2020), only a few studies have demonstrated that bombing is essential for the successful defence of bombardier beetles against predators (Sugiura & Sato, 2018). To test the effectiveness of bombing, it is necessary to use control beetles that can eject hot chemicals and treated beetles that cannot (Sugiura & Sato, 2018). Clarifying the importance of bombing would contribute to understanding the evolution of chemical defence mechanisms in bombardier beetles.

Bombardier beetles can successfully defend themselves against insectivorous animals such as toads, birds, and arthropods (Eisner, 1958, 2003; Eisner & Meinwald, 1966; Eisner & Dean, 1976; Dean, 1980a; Conner & Eisner, 1983; Eisner et al., 2006; Sugiura & Sato, 2018; Sugiura, 2018; Kojima & Yamamoto, 2020). Individuals of some vertebrate species are able to consume bombardier beetles (Dean, 1980a; Sugiura, 2018; Sugiura & Sato, 2018; Kojima & Yamamoto, 2020), whereas several invertebrate species such as spiders always reject them (Eisner & Dean, 1976; Eisner et al., 2006). Therefore, bombardier beetles may deter invertebrate predators more effectively than vertebrate predators. Testing this hypothesis would allow us to identify the types of predators that impose selective pressure on the evolution of anti-predator defences in bombardier beetles.

The bombardier beetle *Pheropsophus jessoensis* Morawitz (Brachininae: Brachinini) is common in farmland, grassland, and forest edges in East Asia, including Japan (Habu & Sadanaga, 1965; Ueno, Kurosawa & Sato, 1985; Yahiro et al., 1992; Ishitani & Yano, 1994; Fujisawa, Lee & Ishii, 2012; Ohwaki, Kaneko & Ikeda, 2015), South Korea (Jung et al., 2012), and China (Li et al., 2012). Like other bombardier species, *P*. *jessoensis* discharges quinones (1,4-benzoquinone and 2-methyl-1,4-benzoquinone) at a temperature of approximately 100 °C when stimulated (Video S1; Kanehisa & Murase, 1977; Kanehisa, 1996). Studies have tested how *P*. *jessoensis* can defend against toads (Sugiura & Sato, 2018), frogs (Sugiura, 2018), and birds (Kojima & Yamamoto, 2020). Adult *P*. *jessoensis* were easily swallowed by the toads *Bufo japonicus* Temminck & Schlegel and *Bufo torrenticola* Matsui (Sugiura & Sato, 2018). However, the swallowed *P*. *jessoensis* ejected chemicals inside the toad bodies causing 34.8% of the *B*. *japonicus* and 57.1% of the *B*. *torrenticola* to vomit 12–94 and 15–107 min after being swallowed, respectively (Sugiura & Sato, 2018). Sugiura & Sato (2018) used treated *P*. *jessoensis* that could not eject hot chemicals to show that bombing is essential for the successful escape of *P*. *jessoensis* from toads. Adult *P*. *jessoensis* were also rejected by the pond frog *Pelophylax nigromaculatus* (Hallowell) (Anura: Ranidae) (Sugiura, 2018) and quail *Coturnix japonica* Temminck & Schlegel (Galliformes: Phasianidae) (Kojima & Yamamoto, 2020). However, most of the frogs and quails rejected *P*. *jessoensis* adults before being bombed, suggesting that bombing is not essential for the successful defence of *P*. *jessoensis* against attacks by frogs and quails (Sugiura, 2018; Kojima & Yamamoto, 2020). Therefore, adult *P*. *jessoensis* can effectively defend themselves against vertebrate predators. However, the effectiveness of *P*. *jessoensis* defences against invertebrate predators remains unexplored.

In this study, I investigated the defence of *P*. *jessoensis* against praying mantises (Insecta: Mantodea) under laboratory conditions. Praying mantises are sit-and-wait (ambush) predators that attack various arthropods (Reitze & Nentwig, 1991) and small vertebrates (Nyffeler, Maxwell & Remsen, 2017; Valdez, 2020). Mantises recognise prey by movement and catch them using their raptorial forelegs (Rilling, Mittelstaedt & Roeder, 1959; Corrette, 1990). Mantises have powerful mouthparts and can devour tough prey (Reitze & Nentwig, 1991). Although praying mantises have been used to investigate the effectiveness of anti-predator defences in many insect species (Berenbaum & Miliczky, 1984; Reitze & Nentwig, 1991; Honma, Oku & Nishida, 2006; Whitman & Vincent, 2008; Rafter, Agrawal & Preisser, 2013; Mebs, Yotsu-Yamashita & Arakawa, 2016; Mebs et al., 2017; Rafter et al., 2017a, 2017b; Mebs, Wunder & Toennes, 2019; Prudic et al., 2019), only one study has used the mantis as a model predator of bombardier beetles. Eisner (1958) provided an adult female mantis [*Hierodula patellifera* (Audinet-Serville) (Mantidae)] with three adult bombardier beetles (*Brachinus tenuicollis* LeConte) under laboratory conditions; two of the three beetles successfully defended themselves against the mantis, while the mantis ate the third. Because the sample size was very small, the defence effectiveness of bombardier beetles against mantises remains unclear. To test whether bombardier beetles can effectively defend themselves against praying mantises, I quantified the defensive behaviour of the bombardier beetle *P*. *jessoensis* against three mantis species: *Tenodera sinensis* Saussure, *Tenodera angustipennis* Saussure, and *H*. *patellifera* (all Mantidae). I tested whether bombing is essential for the successful defence of *P*. *jessoensis* against a mantis attack experimentally.

## Materials and Methods

### Study organisms

I collected 60 adult *P*. *jessoensis* from grasslands and forest edges in the Kinki region (Hyogo and Shiga Prefectures) of Japan, in May 2018, May–September 2019, and July–September 2020 (cf. Sugiura & Sato, 2018; Sugiura, 2018). The beetles were kept individually in plastic cases (diameter 85 mm; height 25 mm) with wet tissue paper under laboratory conditions (25 ± 1 °C; Sugiura & Sato, 2018; Sugiura, 2018). Dead *Spodoptera litura* (Fabricius) (Lepidoptera: Noctuidae) larvae were provided as food (Sugiura & Sato, 2018; Sugiura, 2018). Before the experiments, I weighed the beetles to the closest 0.1 mg using an electronic balance (PA64JP; Ohaus, Tokyo, Japan) and measured the body length to the closest 0.01 mm using slide callipers. Beetles were not used repeatedly in different feeding experiments. I conducted the following experiments 57.1 ± 5.0 (range: 5–162) days after collecting the beetles.

I also collected 60 adult mantises (*Tenodera sinensis*, 10 males, 10 females; *Tenodera angustipennis*, 7 males, 13 females; *Hierodula patellifera*, 20 females) from grasslands and forest edges in the Kinki region (Hyogo, Osaka, Japan and Shiga Prefectures) in October 2018, August–October 2019, and September–November 2020 (Sugiura et al., 2019; Sakagami, Funamoto & Sugiura, 2021). In Japan, adult *T*. *sinensis* and *T*. *angustipennis* are common on grasses and herbs at grasslands and forest edges (Watanabe, Miyamoto & Yano, 2013; Sakagami, Funamoto & Sugiura, 2021) where the bombardier beetle *P*. *jessoensis* is also found. Although *H*. *patellifera* adults are also found at forest edges where *P*. *jessoensis* is abundant, this mantis species is arboreal (Watanabe & Yano, 2009; Sakagami, Funamoto & Sugiura, 2021). Therefore, the bombardier beetle species *P*. *jessoensis*, which walks on the ground below grasses and herbs, potentially encounters *T*. *sinensis* and *T*. *angustipennis* adults, but not *H*. *patellifera* adults under field conditions.

Mantises were kept individually in plastic cases (diameter 100 mm; height 100 mm) with wet tissue paper under laboratory conditions (25 ± 1 °C). *Tenebrio molitor* Linnaeus (Coleoptera: Tenebrionidae) and wild-caught insects (e.g., grasshoppers) were provided as food. The mantises were starved for 24 h before the feeding experiments to standardise their hunger level (cf. Sugiura, 2018). I weighed them to the closest 0.1 mg using an electronic balance (PA64JP; Ohaus, Tokyo, Japan) and measured the body length to the closest 0.01 mm using slide callipers. As with the bombardier beetles, individual mantises were not used repeatedly. I conducted the following experiments 11.3 ± 1.5 (range 1–45) days after I collected the mantises.

### Experiments

To test the effectiveness of the anti-predator defences of *P*. *jessoensis* against praying mantises, I conducted the behavioural experiments under laboratory conditions (25 ± 1 °C).

**Figure 1:**
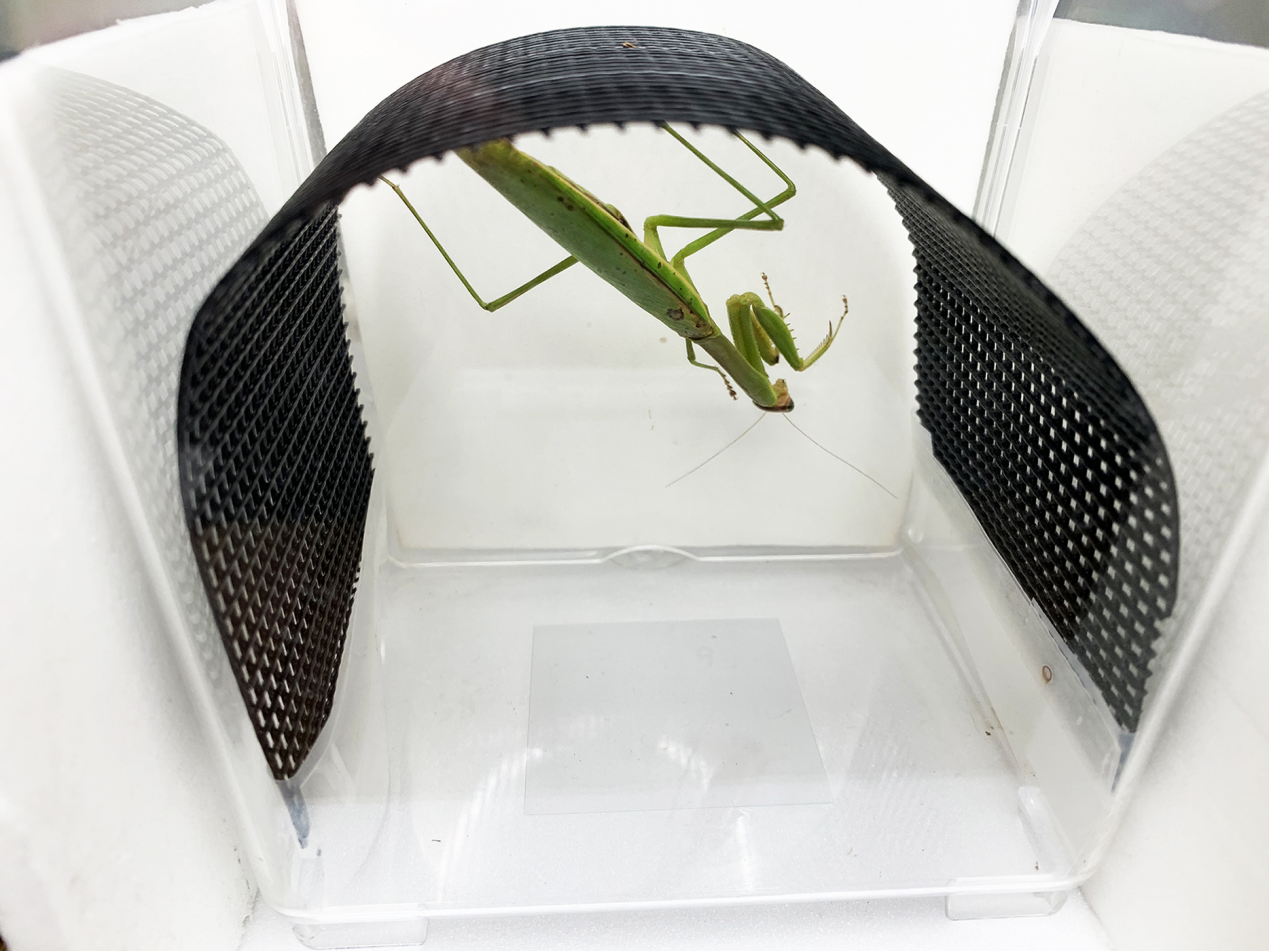
**Experimental arena.** Each mantis hung from a plastic net in a transparent plastic case (length × width × height, 120 × 85 × 130 mm) with its head below its legs. Photo credit: Shinji Sugiura.

First, I placed an adult mantis on a plastic net in a transparent plastic case (length × width × height, 120 × 85 × 130 mm), so that the mantis hung its head down below its legs (Fig. 1). Then, I placed a live *P*. *jessoensis* (‘control’ beetle) on the bottom of the case. Mantises that did not respond to the beetle were not used for the experiments. When a mantis displayed attacking behaviour (i.e., shooting out its forelegs to capture the prey), I recorded the behaviour on video using a digital camera (iPhone XS; Apple Inc., Cupertino, CA, USA) at 240 frames per second. If the mantis rejected the beetle after attacking it, I observed whether the mantis reattacked the same beetle within 1 min. Rejected beetles were also checked for injuries. If a mantis started to eat the beetle, I recorded the feeding time. I also weighed any uneaten beetle parts and calculated the percentage of the beetle eaten. In total, 30 control beetles and 30 mantises (10 *T*. *sinensis*, 10 *T*. *angustipennis*, and 10 *H*. *patellifera*) were used in the experiments.

To test whether the bombing response of *P*. *jessoensis* plays an essential role in deterring a mantis, I provided the mantises with treated *P*. *jessoensis* that were unable to eject hot chemicals. Following the method of Sugiura & Sato (2018), I repeatedly stimulated an adult *P*. *jessoensis* with forceps; the simulated attacks forced them to exhaust their chemicals (i.e., ‘treated’ beetles). Then, I observed whether an adult mantis successfully attacked the treated beetle in a transparent plastic case (length × width × height, 120 × 85 × 130 mm) using the same procedure as for the control beetles. In total, 30 treated beetles and 30 mantises (10 *T*. *sinensis*, 10 *T*. *angustipennis*, and 10 *H*. *patellifera*) were used in the experiments.

All experiments were performed in accordance with Kobe University Animal Experimentation Regulations (Kobe University Animal Care and Use Committee, No. 30–01).

### Data analysis

I used Fisher’s exact test to compare reattack rates between mantis males and females and successful escape rates between control and treated *P*. *jessoensis* from each mantis species and all mantis species combined. I used Student’s *t*-test to compare the body sizes of *P*. *jessoensis* and mantises between the control and treatment experiments. All analyses were conducted using R ver. 3.5.2 (R Core Team, 2018).

## Results

**Figure 2:**
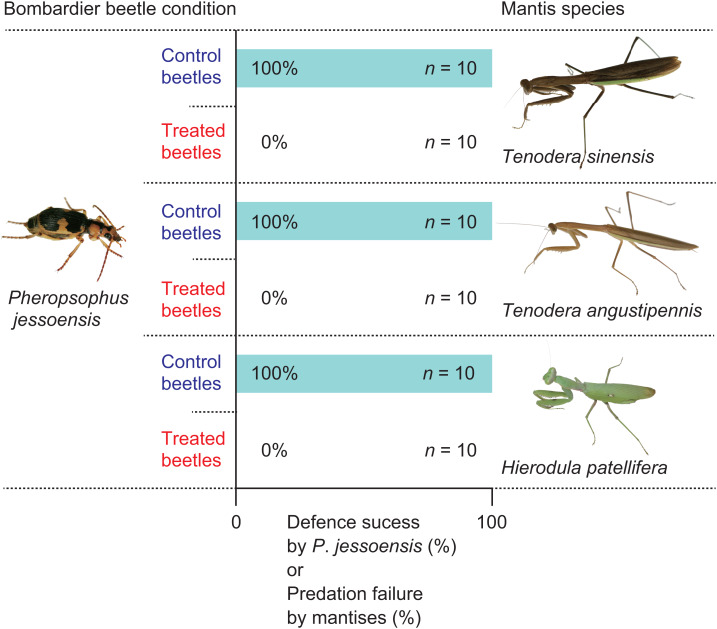
**Defensive success of the bombardier beetle *Pheropsophus jessoensis* and praying mantis predation failure.** Control and treated beetles were *P*. *jessoensis* adults that were able and unable to eject defensive chemicals, respectively. Photo credit: Shinji Sugiura.

**Figure 3:**
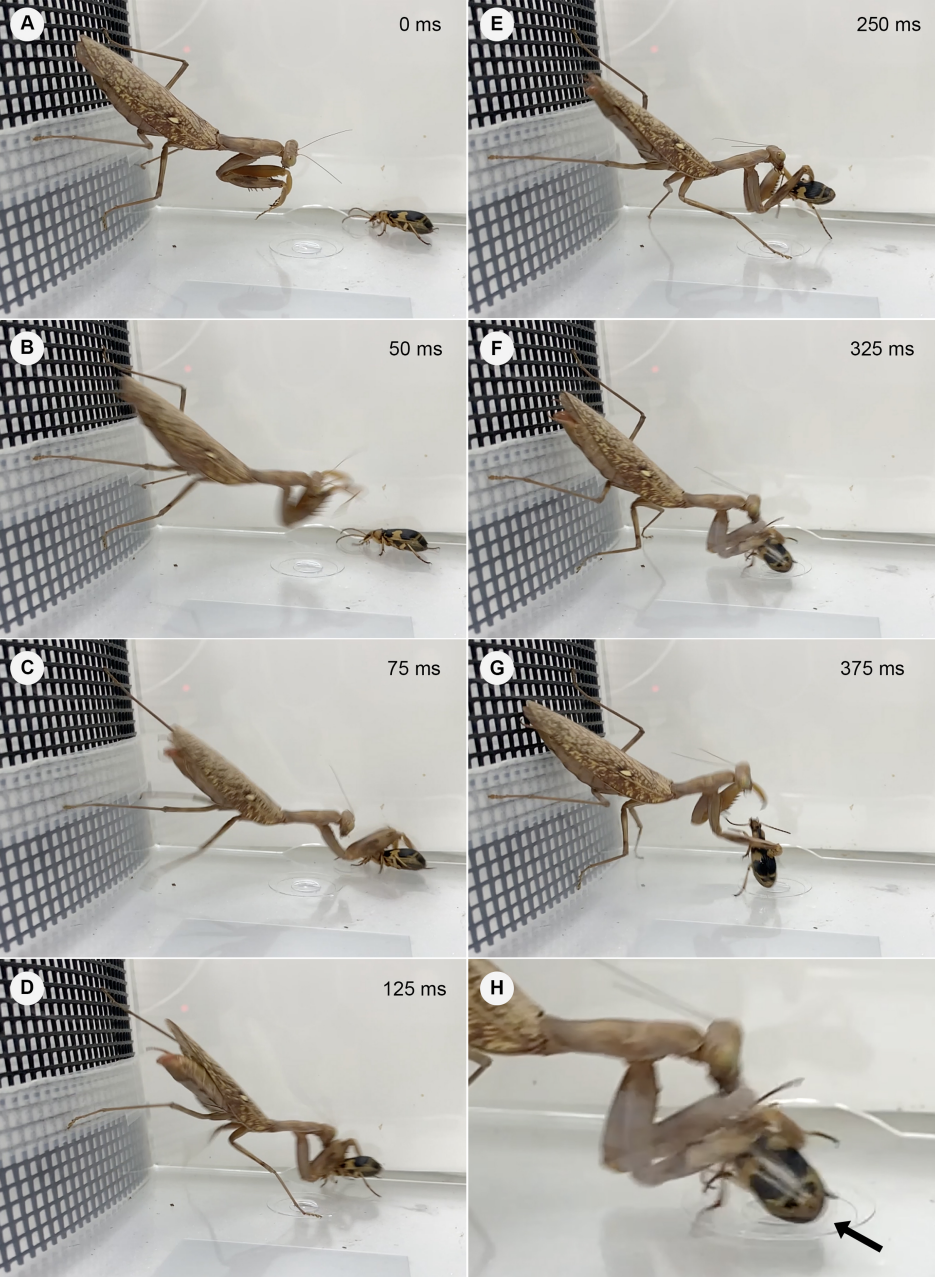
**Temporal sequence of the mantis *Hierodula patellifera* attacking a control adult *Pheropsophus jessoensis***. (A) 0, (B) 50, (C) 75, (D) 125, (E) 250, (F) 325, and (G) 375 ms. (H) Close-up view (F), with the arrow indicating bombing (i.e., the ejected chemicals aimed at the mantis) from the tip of abdomen of the adult *P*. *jessoensis*. The mantis caught the beetle with its raptorial forelegs, but released it immediately after being bombed (see Video S2). Photo credit: Shinji Sugiura.

All mantises used their raptorial forelegs to capture *P*. *jessoensis*. However, all of the control beetles ejected hot chemicals immediately after being captured and the mantises released the beetles immediately after being bombed (*n* = 30; Figs. 2 and 3; Video S2). The chemicals ejected by *P*. *jessoensis* were sprayed on the head, forelegs, and/or thorax of each mantis. In *T*. *sinensis*, 60% of females (*n* = 5) and 20% of males (*n* = 5) reattacked *P*. *jessoensis* within 1 min after releasing them. In *T*. *angustipennis*, 33.3% females (*n* = 6) and 0% of males (*n* = 4) reattacked *P*. *jessoensis*. In *H*. *patellifera*, 20% of females (*n* = 10) reattacked *P*. *jessoensis*. Mantis females reattacked *P*. *jessoensis* more frequently than mantis males; however, these differences were not significant (Fisher’s exact test; *T*. *sinensis*, *P* = 0.5238; *T*. *angustipennis*, *P* = 0.4667; *T*. *sinensis* plus *T*. *angustipennis*, *P* = 0.1571; all species combined, *P* = 0.3742). All reattacking mantises rejected *P*. *jessoensis* again after being bombed. No mantis successfully preyed on control beetles (Fig. 2). After releasing the beetles, all of the mantises were observed to groom the body parts sprayed with hot chemicals. No released *P*. *jessoensis* was injured; all were active (*n* = 30).

**Figure 4:**
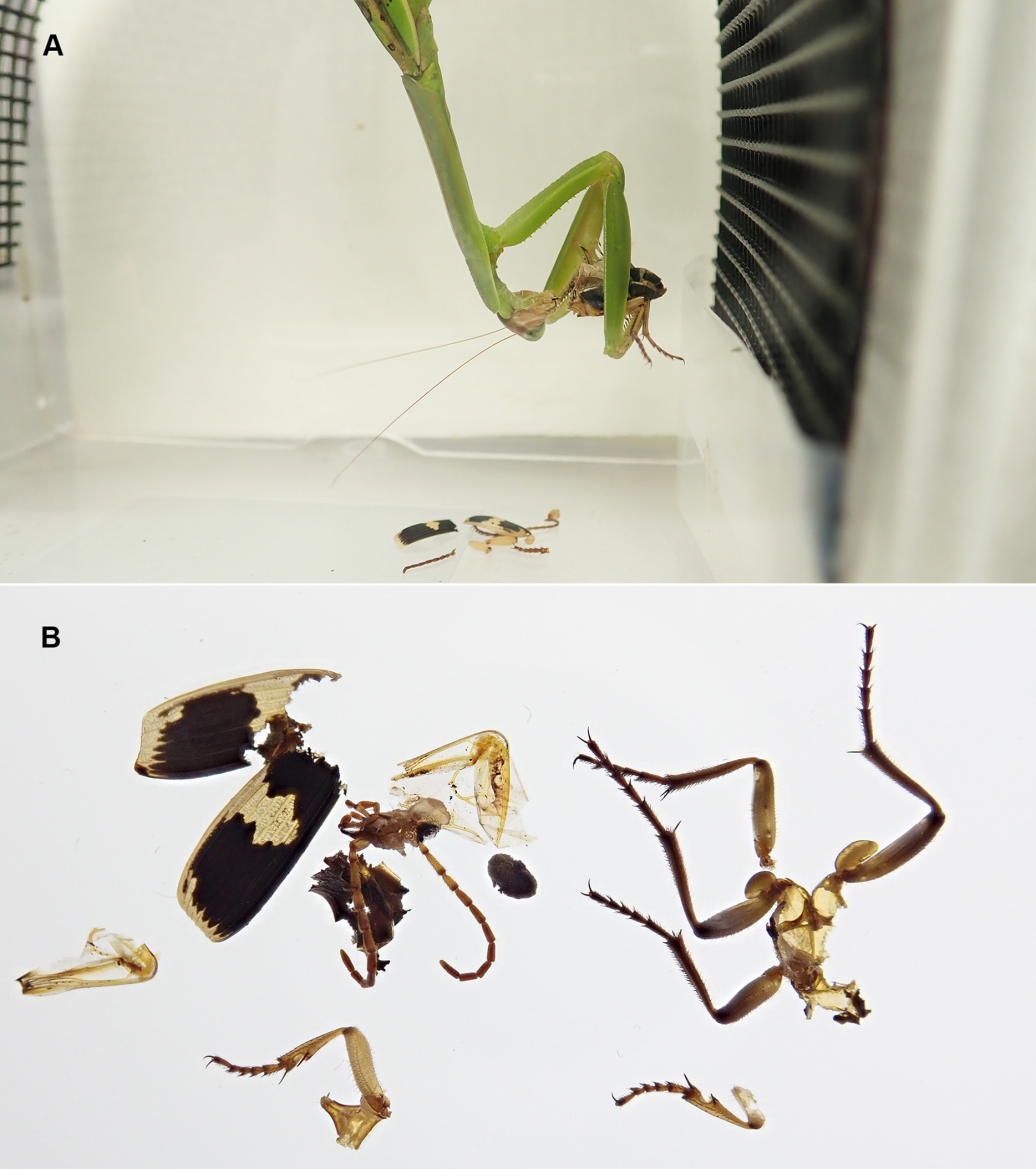
**The mantis *Tenodera angustipennis* feeding on a treated adult *Pheropsophus jessoensis***. (A) Mantis feeding on the beetle body. (B) Leftover antennae, legs, elytra, and hindwings of the beetle. The treated beetle was unable to eject hot chemicals because they had been exhausted by repeated stimulation before the experiment. Photo credit: Shinji Sugiura.

When treated *P*. *jessoensis* that were unable to eject hot chemicals were provided, all the mantises successfully caught the treated beetles using their raptorial forelegs (Video S3). All of the mantises devoured the treated beetles (*n* = 30; Figs. 2 and 4; Video S3). The mantises consumed 90.5% of the body (mainly the thorax and abdomen) of treated *P*. *jessoensis*, while parts of the elytra, legs, and antennae were not eaten (Table 1; Fig. 4). The mean ± standard error feeding time was 52.3 ± 6.6 min (Table 1).

**Table 1:** Consumption of treated *Pheropsophus jessoensis* by praying mantises.

Mantis species	Leftover (mg)	Consumption rate (%)	Feeding time (min)
*Tenodera sinensis*	25.1 ± 4.8	89.0 ± 2.0	56. 0 ± 14.7
*Tenodera angustipennis*	20.2 ± 3.8	90.2 ± 2.2	50.3 ± 8.1
*Hierodula patellifera*	18.1 ± 4.7	92.3 ± 2.0	50.6 ± 11.7
All species combined	21.2 ± 2.6	90.5 ± 1.2	52.3 ± 6.6

**Note:**

Values are the mean ± SE.

The rates of successful escape from mantises significantly differed between the control and treated *P*. *jessoensis* (Fisher’s exact test; *T*. *sinensis*, *P* < 0.0001; *T*. *angustipennis*, *P* < 0.0001; *H*. *patellifera*, *P* < 0.0001; all species combined, *P* < 0.0001). The mean body sizes (lengths and weights) of mantises that attacked control and treated beetles did not differ significantly (Table 2). The mean body sizes (lengths and weights) of control and treated beetles did not differ significantly (Table 2). Therefore, the bombing responses of adult *P*. *jessoensis* deterred mantises of all species, sexes, and sizes.

**Table 2:** Sizes of the bombardier beetle, *Pheropsophus jessoensis*, and praying mantises.

Mantis species	Body size	Treatment		Statistical comparison	
		Control beetles	Treated beetles	*t* value	*P* value
*Tenodera sinensis*	Mantis body length (mm)	80.2 ± 1.8	82.1 ± 1.7	−0.75	0.46
	Mantis body weight (mg)	2234.2 ± 637.3	2273.9 ± 475.7	−0.05	0.96
	Beetle body length (mm)	17.9 ± 0.3	18.2 ± 0.4	−0.58	0.57
	Beetle body weight (mg)	252.1 ± 17.6	229.6 ± 13.9	1.01	0.33
*Tenodera angustipennis*	Mantis body length (mm)	76.3 ± 2.2	79.1 ± 1.7	−0.98	0.34
	Mantis body weight (mg)	1622.4 ± 333.2	1829.3 ± 243.2	−0.50	0.62
	Beetle body length (mm)	17.1 ± 0.5	17.9 ± 0.5	−1.19	0.25
	Beetle body weight (mg)	216.0 ± 19.5	222.6 ± 21.7	−0.23	0.82
*Hierodula patellifera*	Mantis body length (mm)	58.3 ± 0.6	56.2 ± 0.9	1.83	0.09
	Mantis body weight (mg)	1736.4 ± 120.0	1850.0 ± 108.6	−0.70	0.49
	Beetle body length (mm)	17.6 ± 0.5	17.7 ± 0.5	−0.07	0.94
	Beetle body weight (mg)	247.7 ± 23.1	225.9 ± 20.0	0.71	0.49
All species combined	Mantis body length (mm)	71.6 ± 2.0	72.4 ± 2.3	−0.27	0.79
	Mantis body weight (mg)	1864.3 ± 239.6	1984.4 ± 179.4	−0.40	0.69
	Beetle body length (mm)	17.5 ± 0.3	17.9 ± 0.3	−1.08	0.28
	Beetle body weight (mg)	238.6 ± 11.6	226.0 ± 10.5	0.80	0.43

**Note:**

Values are the mean ± SE.

## Disussion

Some praying mantises can prey on well-defended insects, although the predation success rate varies among prey insect species (Reitze & Nentwig, 1991). The effectiveness of chemical defences by bombardier beetles against mantises remains unclear (Eisner, 1958). In this study, I tested the effectiveness of the defences of the bombardier beetle, *P*. *jessoensis*, against three mantis species under laboratory conditions (Fig. 2). My experiments demonstrated that bombing was essential for successful defence by *P*. *jessoensis* against mantises, which were always deterred (Fig. 2). To my knowledge, this is the first study to document a perfect defence against praying mantises by insects smaller than mantises.

Dean (1980b) experimentally investigated the relative importance of the toxic chemicals and heat produced by bombing for the successful defence of bombardier beetles against toads. Although the combination of chemicals and heat played an important role in deterring toads, the chemicals served as the primary defence and bombing as a secondary defence (Dean, 1980b). Toxic chemicals or other material on the body of *P*. *jessoensis* functioned as a primary deterrent against frogs (Sugiura, 2018) and birds (Kojima & Yamamoto, 2020), suggesting that bombing is not essential for the successful defence of *P*. *jessoensis* against frogs and birds. However, all praying mantises consumed the treated *P*. *jessoensis* (Fig. 2), suggesting that chemicals on the body of *P*. *jessoensis* could not deter mantises. Studies have indicated that chemically defended arthropods could not effectively deter mantises (Reitze & Nentwig, 1991). For example, mantises such as *T*. *sinensis* could consume toxic caterpillars after removing (‘gutting’) the midgut containing toxic plant material (Rafter, Agrawal & Preisser, 2013; Mebs et al., 2017; Mebs, Wunder & Toennes, 2019). Several mantis species could also tolerate noxious chemicals such as tetrodotoxin, cardenolides, and quinine used as anti-predator defences by toxic arthropods (Mebs, Yotsu-Yamashita & Arakawa, 2016; Mebs et al., 2017; Rafter et al., 2017a, 2017b; Mebs, Wunder & Toennes, 2019). Therefore, bombing plays an essential role in defending against mantis predation, although additional experiments are needed to test the importance of heat in the successful defence of *P*. *jessoensis* against mantises.

Some predators avoid attacking bombardier beetles after experiencing the toxic chemicals (Dean, 1980a; Kojima & Yamamoto, 2020). Dean (1980a) found that many American toads, *Anaxyrus americanus* (Holbrook) (Anura: Bufonidae), did not reattack bombardier beetles (*Brachinus* spp.) for at least 30 min after rejecting them. Kojima & Yamamoto (2020) observed that some quail exposed to live *P*. *jessoensis* avoided them for up to 5 weeks. In this study, 26.7% of mantises reattacked *P*. *jessoensis* within 1 min after being bombed; *P*. *jessoensis* mantises were reattacked more frequently by females than by males, although these differences were not significant. Hungrier mantises (starved >24 h) may be more likely to reattack *P*. *jessoensis* after being bombed. However, *P*. *jessoensis* should be capable of easy escape from mantises before they reattack under field conditions, because *P*. *jessoensis* can rapidly leave the site after release.

Chemically defended prey produce toxic chemicals that force predators to spit them out (Taniguchi et al., 2005; Whitman & Vincent, 2008; Matsubara & Sugiura, 2017). However, the first predator attack potentially damages the defended prey. Therefore, the chemically defended prey may have evolved tolerance for predator biting and other attacks (Sugiura & Sato, 2018; Sugiura, 2020a). In this study, none of the *P*. *jessoensis* released by mantises were injured, suggesting that *P*. *jessoensis* has a body tough enough to survive an attack by the raptorial forelegs of mantises.

## Conclusions

While the hot chemicals ejected by bombardier beetles deter some vertebrate species, these species do not always reject the bombardier beetles; some individuals are able to consume the beetles (Dean, 1980a; Sugiura, 2018; Sugiura & Sato, 2018; Kojima & Yamamoto, 2020). Eisner & Dean (1976) reported that all individuals of an orb-weaving spider species *Trichonephila clavipes* (Linnaeus) rejected bombardier beetles (*Brachinus* spp.). Eisner et al. (2006) showed that the bombardier beetle *Pheropsophus aequinoctialis* (Linnaeus) always deterred the wolf spider *Schizocosa ceratiola* (Gertsch & Wallace) (Araneae: Lycosidae). In this study, *P*. *jessoensis* bombing always deterred praying mantises. Therefore, hot chemicals discharged by bombardier beetles may deter arthropod predators more effectively than vertebrate predators.

## Supplemental Information

10.7717/peerj.11657/supp-1 Supplemental Information 1 An adult *Pheropsophus jessoensis* bombing. The beetle ejected hot chemicals when stimulated with forceps. This is the video in Sugiura (2018). Video credit: Shinji Sugiura.

10.7717/peerj.11657/supp-2 Supplemental Information 2 A praying mantis, *Hierodula patellifera*, attacking a control adult *Pheropsophus jessoensis*. The mantis caught the beetle using its raptorial forelegs but released it immediately after being bombed. Video credit: Shinji Sugiura.

10.7717/peerj.11657/supp-3 Supplemental Information 3 A praying mantis attacking a treated adult *Pheropsophus jessoensis*. The mantis caught this beetle using its raptorial forelegs and ate it. The treated beetle was unable to eject hot chemicals because they had been exhausted by repeated stimulation before the experiment. Video credit: Shinji Sugiura.

## Additional Information and Declarations

### Field Study Permissions

The following information was supplied relating to field study approvals (i.e., approving body and any reference numbers): My study was not conducted in any national parks or protected areas. Study insects were not protected species; no specific permissions are required to collect non-protected insects in non-protected areas in Japan.

### Data Availability

The following information was supplied regarding data availability: The raw data are available at figshare: Sugiura, Shinji (2021): Data from: Beetle bombing always deters praying mantises. figshare. Dataset. DOI 10.6084/m9.figshare.14443748.v1.
